# The AMP-Activated Protein Kinase Plays a Role in Antioxidant Defense and Regulation of Vascular Inflammation

**DOI:** 10.3390/antiox9060525

**Published:** 2020-06-16

**Authors:** Thomas Jansen, Miroslava Kvandová, Andreas Daiber, Paul Stamm, Katie Frenis, Eberhard Schulz, Thomas Münzel, Swenja Kröller-Schön

**Affiliations:** 1 Center for Cardiology, Department of Cardiology 1—Molecular Cardiology, University Medical Center, Johannes Gutenberg University, 55131 Mainz, Germany; thomas.jansen@unimedizin-mainz.de (T.J.); Miroslava.Kvandova@unimedizin-mainz.de (M.K.); paul.stamm@unimedizin-mainz.de (P.S.); kfrenis@students.uni-mainz.de (K.F.); tmuenzel@uni-mainz.de (T.M.); 2 Partner Site Rhine-Main, German Center for Cardiovascular Research (DZHK), Langenbeckstr. 1, 55131 Mainz, Germany; 3 Department of Cardiology, Allgemeines Krankenhaus Celle, 29223 Celle, Germany; eberhard.schulz@akh-celle.de

**Keywords:** AMP-activated protein kinase, vascular inflammation, oxidative stress, endothelial dysfunction, hypertension

## Abstract

Cardiovascular diseases represent the leading cause of global deaths and life years spent with a severe disability. Endothelial dysfunction and vascular oxidative stress are early precursors of atherosclerotic processes in the vascular wall, all of which are hallmarks in the development of cardiovascular diseases and predictors of future cardiovascular events. There is growing evidence that inflammatory processes represent a major trigger for endothelial dysfunction, vascular oxidative stress and atherosclerosis and clinical data identified inflammation as a cardiovascular risk factor on its own. AMP-activated protein kinase (AMPK) is a central enzyme of cellular energy balance and metabolism that has been shown to confer cardio-protection and antioxidant defense which thereby contributes to vascular health. Interestingly, AMPK is also redox-regulated itself. We have previously shown that AMPK largely contributes to a healthy endothelium, confers potent antioxidant effects and prevents arterial hypertension. Recently, we provided deep mechanistic insights into the role of AMPK in cardiovascular protection and redox homeostasis by studies on arterial hypertension in endothelial and myelomonocytic cell-specific AMPK knockout (Cadh5CrexAMPKfl/fl and LysMCrexAMPKfl/fl) mice. Using these cell-specific knockout mice, we revealed the potent anti-inflammatory properties of AMPK representing the molecular basis of the antihypertensive effects of AMPK. Here, we discuss our own findings in the context of literature data with respect to the anti-inflammatory and antioxidant effects of AMPK in the specific setting of arterial hypertension as well as cardiovascular diseases in general.

## 1. Introduction

Cardiovascular diseases are the leading cause of global deaths and life years spent with a severe disability. The most recent report from the World Health Organization revealed an annual loss of life of 17 million due to cardiovascular diseases, and further, that these deaths could be significantly decreased by reduction in tobacco use and mitigation of other lifestyle parameters. (e.g., diet and physical activity) [1]. All these main risk factors cause vascular oxidative stress resulting in endothelial dysfunction and the activation of inflammatory processes, both of which are early precursors of atherosclerotic changes in the vascular wall. As such, oxidative stress, endothelial dysfunction and active inflammation are real hallmarks of the development of cardiovascular diseases and predictors of future cardiovascular events [2,3].

**Figure 1:**
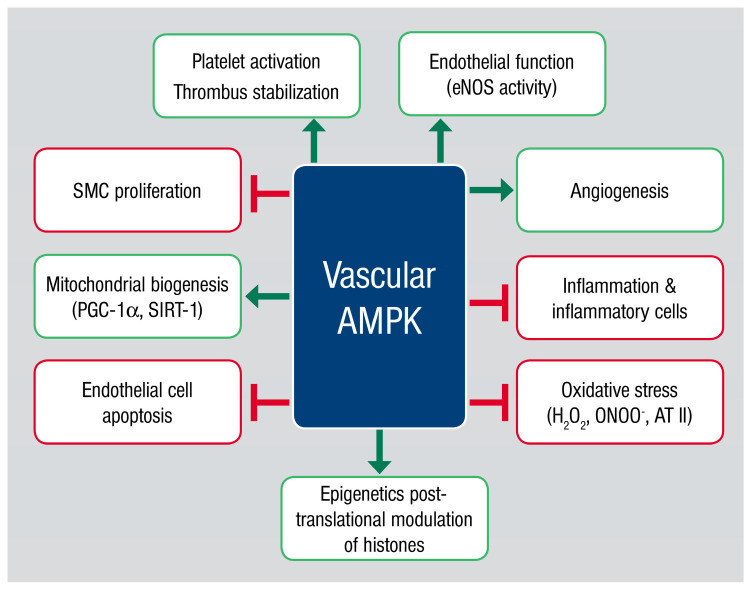
Physiological functions of the vascular adenosine monophosphate-activated protein kinase (AMPK). Activation of the vascular AMPK leads to enhanced platelet activation and thrombus stabilization, beneficial impact on the vascular function by eNOS phosphorylation and reduction of oxidative stress by controlling mitochondrial biogenesis and mitochondrial gene expression. Furthermore, it is known that inflammatory processes as well as the recruitment of inflammatory cells into the vascular wall are regulated by AMPK. In this context, AMPK is able to promote angiogenesis, reducing endothelial cell apoptosis and inhibiting smooth muscle cell proliferation. The most recent data points to functions of AMPK regulating the posttranslational modulation of histones leading to changes in epigenetic regulation of gene expression. Abbreviations: AMPK: adenosine monophosphate-activated protein kinase; AT II: angiotensin II; eNOS: endothelial nitric oxide synthase; H_2_O_2_: hydrogen peroxide; ONOO^−^: peroxynitrite anion; PGC-1α: peroxisome proliferator-activated receptor gamma coactivator 1-alpha; SIRT-1: sirtuin 1; SMC: smooth muscle cell.

Adenosine monophosphate-activated protein kinase (AMPK) was first described in 1988 by Hardie et al. [4,5], who defined this protein as a regulating enzyme of acetyl-CoA-carboxylase and HMG-CoA-reductase. They named this enzyme for its most prominent physiological activator adenosine monophosphate, AMP [5]. Following the elucidation of AMPK’s energy sensing ability in regulating glucose uptake [6] and securing cellular survival under stress conditions [7], the enzyme became the focus of increased research interest. The function of AMPK as an energy sensor is not just restricted to only intracellular processes, even extracellular energy expenditure is balanced by this heterotrimeric kinase. Once activated, AMPK phosphorylates several downstream targets [6,8,9,10], thereby inhibiting energy consumption and promoting energy production to ensure cellular survival [7]. Besides its role as a central hub in cellular energy utilization, AMPK is also involved in a number of other important biological functions (for review see Kahn et al., [11]), including the recently described regulation of the epigenetic pathways (Figure 1) [11,12]. The first hints of AMPK’s cardiovascular influence were shown in 1999 by Chen et al. who reported direct phosphorylation sites on endothelial NO-Synthase enzyme in endothelial cells and myocytes [13]. The present review focuses on the evidence that AMPK represents a central regulator of vascular inflammation by maintaining the oxidative homeostasis in the cell.

## 2. AMPK Structure, Activation Process, Targets and Pathways

**Figure 2:**
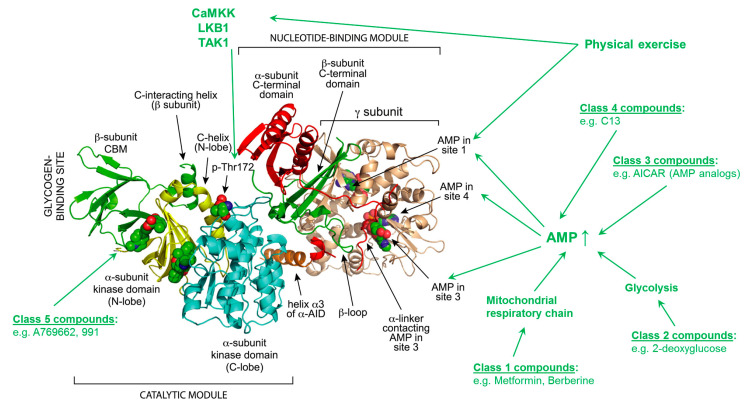
Structure and regulation of heterotrimeric AMPK. The model was created with MacPyMol using protein database (PDB) file 4CFE. The α, β and γ subunits are shown in “cartoon view” with domains color-coded. The three bound molecules of AMP, and phospho-Thr172 are shown in “sphere view” with C atoms green, O red, N blue and P orange. The different classes of compounds and their specific points of action are listed in green. Abbreviations: AICAR: 5-aminoimidazole-4-carboxymide ribonucleoside; AID: autoinhibitory domain; AMP: adenosine monophosphate; AMPK: adenosine monophosphate protein kinase; CaMKK: calmodulin-dependent kinase kinases; CBM: carbohydrate-binding module; C13: compound 13; LKB1: liver kinase B1; TAK1: transforming growth factor-β-activated kinase 1; Thr: threonin; adopted from [59] with permission. Copyright © 2014 Elsevier Inc. All rights reserved.

AMPK is a conserved serine/threonine heterotrimeric kinase, which is known to be a cellular energy sensor. AMPK isoforms are assembled as heterotrimeric complexes that include one catalytic α subunit and two regulatory β and γ subunits (Figure 2) [8]. These different isoforms of subunits (α1/α2, β1/β2, γ1/γ2/γ3) are encoded by separate genes. In total, there are 12 isoforms specifically distributed by cell type according to their unique function [14]. The α subunit contains the kinase domain at its N-terminus, whereas the C-terminal region binds to the ß subunit [15]. The important fuel sensing, carbohydrate-binding domain is located in the β subunit which is also responsible for the interaction with the γ subunit [16]. The individual activation processes take place in a number of domains located in the γ subunit [15]. AMPK activation is delineated in three steps based on the AMP/ATP ratio: phosphorylation by upstream protein kinases, dephosphorylation processes by upstream protein phosphatases and then allosteric activation by AMP or/and ATP. All of these mechanisms ensure that the system is sensitive and responds to small AMP/ATP ratio changes [17,18]. AMP binding and phosphorylation processes cause conformational changes after phosphorylation/dephosphorylation at Thr172 [18,19]. The mechanism of Thr172 phosphorylation and consequent nucleotide binding is regulated by two individual upstream kinases, liver kinase B1 (LKB1) [20,21] and calcium/calmodulin-dependent protein kinase 2 (CaMMK2) [17]. CaMMK2 activation is dependent on intracellular calcium levels and is the leading activator of AMPK in specific cell types [22], including endothelial cells [23], hypothalamic neurons [24] and T-cells [25]. LKB1, in contrast to CaMMK2, is permanently active [17] and known as a master upstream kinase of AMPK and other kinases [22]. The potential for activation by these two master kinases puts AMPK in the position to receive information from several cellular signaling pathways [17].

Pharmacological activation of AMPK can be achieved through the use of five classes of compounds (Figure 2), divided by the activation mechanism [14]. The compounds in class 1 (e.g., berberine or metformin) inhibit the complex I of the mitochondrial respiratory chain, whereas class 2 compounds (e.g., 2-deoxyglucose) inhibit glycolysis [15]. In class 3, AICAR (5-aminoimidazole-4-carboxymide ribonucleoside), widely used as an AMPK activator, is converted to the AMP analog ZMP and mimics the activation process by AMP [26,27]. In class 4, C13 is listed as a specific activator of the α1 subunit. This compound is internalized in the cell, converted to the AMP analog C2 and binds to the γ subunit [28,29,30]. Among the specific AMPK activators, A769662 represents a small molecule that activates AMPK via an allosteric action. Mechanistically, dephosphorylation of active αAMPK at Thr172 is prevented by a reciprocal action with the β1-subunit. Class 5, the last group of activators, are synthetic compounds (e.g., MT63-78, PF-249) which bind at the ADaM site in the β subunit of the AMPK complex [26,31,32,33,34].

A wide range of endogenous physiological stimuli has been found to activate AMPK in the vasculature, including angiotensin II [35], hypoxia [36], shear stress [37], low glucose [38] and oxidative stress [39,40]. Vasoactive substances like thrombin [23], VEGF [41], bradykinin [42] and estrogen [43] also activate AMPK through a CAMKK2-mediated pathway. Additionally, a number of cholesterol-lowering [44,45] and anti-diabetic [46,47,48,49] drugs activate AMPK, which is thought to contribute to their beneficial effects on the cardiovascular system.

Reactive oxygen species may also play a role in the activation of AMPK independently of adenine nucleotides [50,51,52,53]. Several groups have reported AMPK activation in the form of oxidative changes to Cys299 and Cys304 in the catalytic subunit [54] in response to increased concentrations of exogenous H_2_O_2_ [35]. These alterations in AMPK’s activity were independent of adenine nucleotides, leading to the hypothesis that AMPK is redox-sensitive [54]. Other regulatory pathways that are related to redox signaling and oxidative stress are based on alterations by SIRT6 [55], as well as the antioxidant and epigenetic drug resveratrol [56].

Though there are numerous activator compounds, there is no specific pharmacological inhibitor of the AMPK complex. The compound C is the only known pharmacological inhibitor, but it is not a specific inhibitor [57]. Thus, to investigate the effects mediated by AMPK inhibition, it is necessary to employ genetic approaches, such as knockout models (global or tissue-/cell-specific) or siRNA/microRNA, among others [58].

## 3. AMPK in Vascular Oxidative Stress

Endothelial function is predicted by the rate of vascular NO production and simultaneous consumption by reactive oxygen species (ROS). ROS, like the superoxide anion (O_2_^•−^), hydrogen peroxide (H_2_O_2_) and hydroxyl radical (^•^OH), are produced physiologically by normal cellular metabolic processes. The predominant source of ROS production during metabolic processes is the mitochondrial electron transport chain, especially Complexes I and III. Within these complexes, a reduction of molecular oxygen by the nicotinamide adenine dinucleotide phosphate (NADPH) oxidases (NOX) produces O_2_^•−^ [60,61]. Furthermore, NADH-Dehydrogenase in Complex I is also a critical ROS-generating part of the respiratory chain, which could be a useful indicator of the redox state under physiological conditions [54]. Mitochondria are indeed one of the primary sources of intracellular ROS production and are responsible for up to 95% of total ROS levels [62]. Therefore, a system is needed which is able to maintain the redox homeostasis in both normal and stress conditions. Nuclear factor erythroid 2-related factor 2 (Nrf-2) is a master regulator of the antioxidant and anti-inflammatory defense machinery, participating in the maintenance of the cellular redox balance [63]. The ROS-dependent activation of Nrf-2 under normal or stress conditions can be understood as a stress response to secure oxidative homeostasis [64]. The first change in Nrf-2 activity upon conditions of oxidative stress is driven by the phosphorylation of Ser40 by protein kinase Cδ (PKCδ) leading to the release of Nrf-2 from the Nrf-2-Keap-1 complex. The detachment and resulting stabilization of Nrf-2 in the cytosol allows translocation to the nucleus where it binds to antioxidant response element (ARE) regions, stimulating the expression of antioxidant genes [65,66]. This activation process is also mainly regulated by oxidation of the thiol groups in the Keap-1/Nrf2 complex to induce conformational changes allowing subsequent phosphorylation by PKC. Nrf-2 is a highly regulated protein not just in the cytosol, but also in the nucleus. Nuclear Nrf-2 is regulated by the glycogen synthase kinase 3ß and its downstream target Fyn to induce the nuclear export and final degradation of the protein [67]. AMPK is known to be a positive regulator of Nrf-2 by direct phosporylation on Ser550, leading to accumulation in the nucleus to induce the expression of proteins belonging to the antioxidant defence system of the cell [68].

This direct interaction between AMPK and Nrf-2 points to a critical crosstalk between major regulators of metabolism and the redox system of the cell maintaining the necessary homeostasis to secure cellular survival [68]. In addition to this direct redox regulation of AMPK by oxidation of critical cysteine residues [50,51,52], indirect redox signaling/regulation has also been reported. Sobotta et al. illustrated that cytosolic AMPK can be directly activated by reversible thiol oxidation, e.g., peroxiredoxin dimerization [69], or indirectly by redox-sensitive upstream kinases [70,71]. A factor impairing the redox balance is glucose starvation leading to an elevation of intracellular ROS, where activation of AMPK seems to have a critical role in promoting cellular survival [72]. Taken together, AMPK contributes to signal pathways that regulate the redox balance of the cell determining the emergence of cardiovascular diseases [73].

## 4. AMPK in Endothelial Function

The major factor for the conservation of vascular homeostasis is an intact endothelium, which depends on endothelial nitric oxide synthase (eNOS)-derived NO production. NO is formed by a family of proteins called nitric oxide synthases (NOS) by oxidizing L-arginine under the consumption of molecular oxygen. Endothelium-derived NO is the key player in the regulation of the vascular tone [74,75]. In addition to relaxing vessels, NO exerts anti-inflammatory and antithrombotic effects in the vasculature [76,77,78]. A reduction in the nitric oxide bioavailability leads to endothelial dysfunction which results in hypertension and atherosclerosis [79,80]. There is a myriad of known regulatory mechanisms that affect eNOS activity: including post-translational modifications, availability of cofactors like BH4, protein–protein interactions, prosthetic groups, calcium/calmodulin and several phosphorylation sites [81]. Shear stress, statins and adiponectin are physiological, pharmacological and hormonal stimuli that are known to increase AMPK activity directly and lead to the phosphorylation of eNOS at Ser633/635, increasing the NO bioavailability and/or the antioxidant potential of endothelial cells [74,82,83,84,85,86]. In contrast, inhibition of AMPK—genetically or pharmacologically—decreases the phosphorylation of eNOS at Ser633/635, resulting in impaired NO production [74]. In addition, the group of Fleming and Fisslthaler showed that AMPK is also responsible for the phosphorylation of eNOS at Thr495, which is known to be an inhibitory phosphorylation site [87]. These AMPK-dependent phosphorylation sites of eNOS seem to play a minor role in vivo, suggesting that cardiovascular protection occurs through other pathways, such as the increased bioavailability of NO resulting from improved mitochondrial function (via PGC-1α) or regulation of transcriptional changes of antioxidant and anti-inflammatory enzymes like superoxide dismutase 2 (SOD2) [88]. In accordance with this, global deletion of the vascular isoform of AMPK, α1, elicited neither endothelial dysfunction, a decrease in eNOS phosphorylation on Ser1177 nor a consequent decrease in NO bioavailability [39].

As eNOS is a strongly regulated enzyme, its phosphorylation status is not the only determinant in its activity. Intracellular tetrahydrobiopterin (BH_4_) levels are essential for dimerization and consequently for the functionality of eNOS [89,90]. Furthermore, BH_4_ levels are determined by a balance in the rate of synthesis, oxidation and recycling of BH_2_ to BH_4_. Production of BH_4_ is regulated by guanosine-5’-triphosphatecyclohydrolase I (GCH I) [91]. AMPK also affects this aspect of eNOS regulation by suppressing the 26S proteasome-dependent GTPCH I degradation in vitro, reversing diabetes-induced endothelial dysfunction [90]. Due to these links to eNOS regulation, the idea of “AMPK activation as a strategy for protecting vascular endothelial function” was already published in 2008 [92].

**Figure 3:**
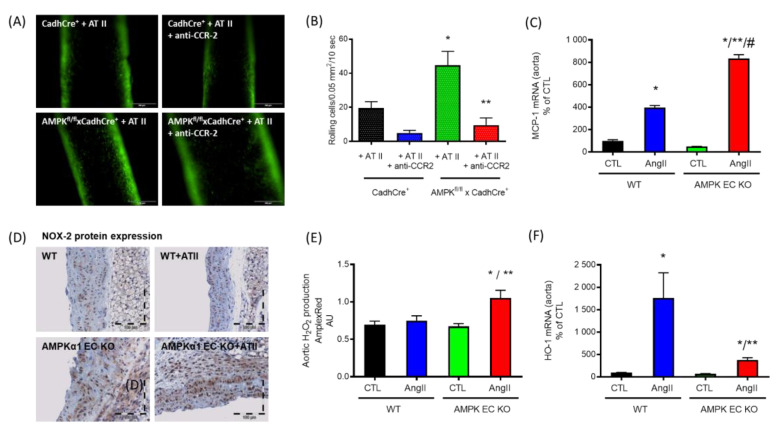
Functions of endothelial specific α1AMPK. (**A**,**B**) Epifluorescence intravital microscopy was used to visualize adherent and rolling leucocytes in carotid arteries. Nucleated cells were stained with acridine orange (green fluorescence). Representative images of carotid arteries are shown after 7 days of continuous AT II infusion (*n* = 8) before and 60 min after single intraperitoneal injection of anti-CCR2 antibody (anti-CCR2). Deletion of endothelial-specific α1AMPK (AMPK^fl/fl^×CadhCre^+^ + AT II) leads to a loss of endothelial barrier function and an enormous AT II-induced increase in CCR-2-dependent recruitment of inflammatory cells to the vascular wall in comparison with the wild-type littermates (CadhCre^+^ + AT II) that was prevented by anti-CCR2 treatment. * *p* < 0.05 vs. CadhCre^+^ + AT II, ** *p* < 0.05 vs. AMPK^fl/fl^×CadhCre^+^ + AT II. (**C**) mRNA expression of MCP-1 was significantly upregulated in aortic tissue of AT II-treated mice lacking α1AMPK in the endothelium (*n* = 6), which was accompanied by immune cell infiltration and (**D**) increased NOX-2 protein measured by immunohistochemistry (brown staining) (*n* = 4). (**E**) Furthermore, oxidative stress, measured by Amplex Red assay (*n* = 4), was significantly increased in mice lacking the endothelial α1AMPK treated with AT II. (**F**) In addition to this, the loss of endothelial α1AMPK impaired the antioxidant defense induced normally by AT II (*n* = 11). * *p* < 0.05 vs. WT CTL, ** *p* < 0.05 vs. WT AngII, # *p* < 0.05 vs. AMPK EC KO CTL. Data were reused from [94] with permission. Copyright © 2019, Springer-Verlag GmbH Germany, part of Springer Nature. Abbreviations: AMPK EC KO: TekCre-specific AMPK deletion (AMPK^fl/fl^xTekCre^+^); AMPK: adenosine monophosphate-activated protein kinase; AT II: angiotensin II; CCR-2: C-C motif chemokine receptor 2; HO-1: heme oxygenase-1; H_2_O_2_: hydrogen peroxide; NOX-2: nicotinamide adenine dinucleotide phosphate oxidase 2; MCP-1: monocyte chemoattractant protein-1.

Our group showed that angiotensin II infusion (1 mg/kg/d) resulted in significant endothelial dysfunction which was characterized by a dramatic increase in the superoxide signal in the vascular wall as well as an increased myocardial NADPH oxidase activity [35]. The activation of AMPK in this model using daily AICAR injections (200 mg/kg/d) led to a significant improvement of the endothelial dysfunction, diminished superoxide production in the vascular wall and a reduction in myocardial NADPH oxidase activation [35]. Based on these data, we demonstrated that AMPK, especially the isoform α1AMPK, is a protective enzyme in the vascular system. Further evidence showed that AICAR injections were ineffective in AT II-infused α1AMPK or PGC-1α global knockout animals [35]. Furthermore, we were able to show that AT II (low dose 0.1 mg/kg/d) infusion in α1AMPK knockout animals resulted in a more pronounced endothelial dysfunction, oxidative stress and inflammatory response compared with the corresponding wild-type animals. These changes correlated with increased expression of the NADPH oxidase subunit NOX-2 (mRNA and protein), which represents the essential catalytic subunit of the phagocytic NADPH oxidase. Suppression of the NADPH oxidase by in vivo administration of apocynin decreased vascular ROS production and also preserved the vascular inflammatory response as assessed by inducible NO synthase (iNOS), cyclooxygenase-2 (COX-2) and vascular cell adhesion molecule 1 (VCAM-1). However, an important limitation of this study was in the use of global knockout animals, as α1AMPK deletion has effects in a variety of tissues that are not isolated only to vascular tissue. Further, Föller et al. were able to show that α1AMPK plays a decisive role in erythrocyte survival through their demonstration that erythrocytes of α1AMPK global knockout animals have a much shorter lifespan, which leads to a severe splenomegaly [93]. Therefore, a new study was designed using endothelial-specific knockout animals, which lack α1AMPK exclusively in endothelial cells, presenting novel mechanistic insights on the protection of the vasculature against oxidative damage in vivo [94]. Loss of endothelial α1AMPK impairs the endothelial cell barrier function, resulting in an enhanced recruitment of inflammatory cells to the vascular wall (Figure 3A,B). Based on these changes, cytokines and vascular adhesion molecules, as well as inflammatory proteins were upregulated. In this endothelial-specific α1AMPK mouse model, we found that the counterpart of the chemokine C-C chemokine receptor type 2 (CCR2), the ligand monocyte chemoattractant protein-1 (MCP-1) (Figure 3C), significantly increased in the aorta of these mice, which resulted in higher α1AMPK-mediated immune cell infiltration and increased NOX-2 protein expression (Figure 3D). This was accompanied by augmented production of vascular reactive oxygen species (Figure 3E). In addition to the inflammatory response, AT II-induced cytoprotective HO-1 upregulation was suppressed in α1AMPK-deficient cells [94]. In summary, endothelial-specific α1AMPK seems to be a crucial element in the recruitment of inflammatory cells to the vasculature, modulating inflammatory responses and vascular function in a pro-oxidative milieu [94].

## 5. AMPK in Vascular Inflammation

Inflammation is triggered in response to tissue injury and represents an energy-consuming process. Here, AMPK has been shown to close the gap between metabolism and inflammation since metabolic control of immune cells determines their fate and function. During recent years, it has become apparent that profound knowledge of metabolic pathways within immune cells is important to complete the picture of the pro- and anti-inflammatory response. In this regard, AMPK is known to have anti-inflammatory properties, reported in diverse animal models of inflammation [95,96]. Among immune cells, experimental work is mainly focused on the role of AMPK in T-cells and macrophages [97,98,99]. The functional role of AMPK in T-cells has been studied intensively, shedding light on α1AMPK’s important role in the proliferation and differentiation of CD8^+^ cytotoxic T lymphocytes. α1AMPK-depleted T-cells showed a striking defect in their ability to generate memory CD8+ T-cell responses during Listeria monocytogenes infection [100]. Alongside this important finding, work by Blagih et al. showed that AMPK couples nutrient availability to T-cell effector function, confirming that α1AMPK was required for the metabolic adaption and following adequate T-cell response in the process of inflammation [101]. Several disease models have been used to uncover important mechanisms beyond AMPK’s physiological functions, especially of the α1 subunit, in the regulation of inflammatory control.

**Figure 4:**
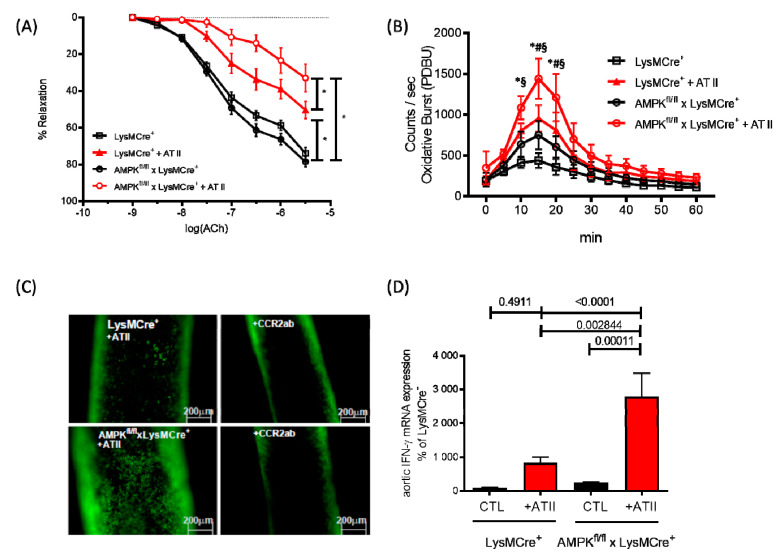
Functions of monocyte-specific α1AMPK. (**A**) Endothelial-dependent relaxation in response to acetylcholine was analyzed by isometric tension studies in intact aortic rings from myelomonocytic cell-specific α1AMPK knockout mice (α1AMPK^fl/fl^ LysM-Cre) and corresponding wild-type littermates (LysMCre/wt) treated with AT II (1.0 mg/kg for 7 days; *n* = 8–10 mice/group). (**B**) Phorbolester (PDBu)-stimulated oxidative burst in whole blood was determined by L-012-enhanced chemiluminescence (*n* = 12). * *p* <0.05 vs LysMCre^+^ CTL, § *p* <0.05 vs. AMPK^fl/fl^ x LysMCre^+^, # *p* <0.05 vs. LysMCre^+^ + AT II. (**C**) Epifluorescence intravital microscopy was used to visualize adherent and rolling leucocytes in carotid arteries. Nucleated cells were stained with acridine orange (green fluorescence). Representative images of carotid arteries after 7 days of continuous AT II infusion (*n* = 8), before and 60 min after single intraperitoneal injection of anti-CCR2 antibody (CCR2ab). (**D**) Pro inflammatory cytokine level of IFN-γ was measured by real-time PCR (*n* = 10). Data were reused from [103] with permission. Copyright © 2018, Oxford University Press. Abbreviations: AMPK: adenosine monophosphate-activated protein kinase; AT II: angiotensin II; CCR-2: C-C motif chemokine receptor 2.

A milestone in the field of immune cell-specific AMPK research was the manuscript by Mounier et al. published in 2013 [102]. They described for the first time the consequences of α1AMPK depletion in myeloid cells in the setting of muscle injury in mice. Macrophages lacking the α1AMPK subunit were dysfunctional, in that they maintained M1 polarization [102]. The number of anti-inflammatory M2 macrophages were decreased, resulting in an impairment of both the release of anti-inflammatory cytokines and phagocytosis of necrotic and apoptotic cells. In this context, we investigated the role of α1AMPK in a model of AT II-induced endothelial dysfunction using a myeloid cell-specific α1AMPK knockout mouse. These animals are characterized by a permanent, non-inducible α1AMPK deletion in myeloid cells and display a pro-inflammatory vascular phenotype in response to specific stimuli (e.g., angiotensin II) [103]. Severe endothelial dysfunction (Figure 4A) and oxidative stress (Figure 4B) with a pronounced F4/80+ macrophage and GR1+ granulocyte infiltration in the vasculature were present in these mice, compared with wild-type control animals. Since CCR2 plays a pivotal role in AT II-induced vascular inflammation (Figure 4C), the increased CCR2 expression on α1AMPK-depleted macrophages caused vascular inflammation with a high secretion of IFN-γ, IL-6 and TNF-α (Figure 4D) [103]. In addition to these reports on disease models, other studies highlighted the physiological role of AMPK in the regulation of immune cells, since the modulation of AMPK activity results in an abolished inflammatory response [104,105,106] and demonstrated a significant reduction in IFN-γ and TNF-α release after AMPK activation with metformin, berberine and AICAR.

Accordingly, AMPK is able to regulate the differentiation of monocytes and the release of inflammatory cytokines in macrophages, leading to the conclusion that AMPK could play an important role in the atherosclerosis-mediated inflammation process through the macrophage-derived foam cell function. Recent work by Chen et al. has shown that the activation of AMPK significantly reduced LOX-1 expression and oxLDL uptake in macrophages, mechanistically by the AMPK-mediated suppression of the NF-kB signaling pathway [107]. Additional work by Zhang et al. supported evidence for a significant role of AMPK in atherosclerosis by showing that activation of AMPK in cultured human endothelial cells resulted in an attenuation of the endothelial pro-inflammatory response, by inhibition of monocyte adhesion and VCAM-1 expression. The NF-kB transcriptional activity was enhanced due to the AMPK-mediated acetylation of p300 HAT activity [108]. Other studies have highlighted the anti-inflammatory properties of AMPK in a model of LPS-induced endothelial inflammation. LPS injection represents a severe sepsis model which is characterized by a strong inflammatory response, triggered by Toll-like receptors 2 and 4. Here, the activation of AMPK with metformin suppressed TLR4 activity [109]. Work by Sun et al. showed that TLR4 protein expression induced by LPS can be prevented by AMPK activation with AICAR in EA.hy926 cells. The activation of AMPK with the alternative activator A769662 resulted in a similar TLR4 modulation. Inhibition of AMPK with the compound C abolished the anti-inflammatory effects, giving evidence for these AMPK-mediated effects [44]. Despite these promising and positive results, the consequences of AMPK activity on the modulation of immune cell function remain uncertain and no data are available in respect to immune cell deficiency, which needs to be addressed in upcoming studies. Further work might focus on the role of AMPK in granulocytes, since these cells display the first response in the cascade of inflammation. However, AMPK is ubiquitously expressed in immune cells and mediates diverse anti-inflammatory responses which might indicate AMPK as a therapeutic target in the future.

## 6. Conclusions

**Figure 5:**
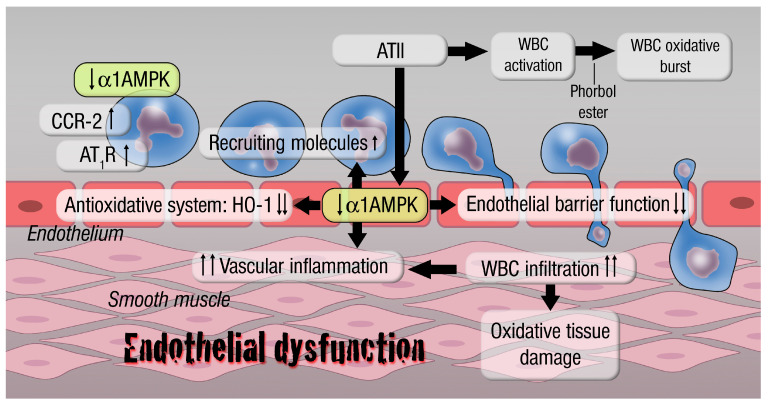
Inflammatory responses on myelomonocytic- and endothelial-specific α1AMPK knockout cells. Loss of myelomonocytic (LysMCre)-specific α1AMPK affects vascular and whole blood oxidative stress and endothelial function by regulating the recruitment of inflammatory cells (enhancement of CCR-2, MCP-1 and other recruiting molecules) to the vascular wall and higher white blood cell (WBC) activation state (reflected by WBC oxidative burst). This also promotes an increase in angiotensin II receptor type 1, leading to higher responsiveness towards pro-oxidant stimuli and in conclusion a pro-inflammatory phenotype (IL-6, TNFα, IFNγ upregulation). Loss of endothelial-specific α1AMPK impairs the endothelial barrier function, resulting in an increased CCR-2/MCP-1 (recruiting molecules)-mediated infiltration of inflammatory cells. This situation provokes an overproduction of reactive oxygen species, leading to oxidative tissue damage and vascular inflammation. Furthermore, deletion of endothelial-specific α1AMPK disables the antioxidant system by blunting HO-1 induction. Abbreviations: AMPK: adenosine monophosphate-activated protein kinase; AT II: angiotensin II; AT_1_R: angiotensin II receptor type 1; CCR-2: C-C motif chemokine receptor 2; HO-1: heme oxygenase-1; WBC: white blood cell.

Over the last decades, increased research interest has been shown in metabolic pathways, especially those involving AMPK, in cardiovascular research. Our understanding of pathologies is continuously improved by more detailed molecular insights into metabolic or inflammatory diseases leading to cardiovascular outcomes, culminating in new therapeutic concepts. Beyond the well-established antioxidant, anti-inflammatory and metabolic protective effects mediated by AMPK, there are also novel pathways in the focus of current and future research detailing the overall cardio-protective effects of AMPK (summarized in Figure 5). The summarized data about the effect of AMPK on cardiovascular diseases are more than promising, but remain limited due to its generation in animal models. Especially the application of cell-specific knockout models provided an important gain of knowledge about the pathophysiologic basis of AMPK-mediated vascular effects. It is necessary to translate these findings in the setting of human disease. An important next step toward accomplishing this translation is to determine the role of α1AMPK in patients with severe hypertension or endothelial dysfunction. In this regard, the AMPK-dependent myeloid cell function should be addressed as well as the T-cell function, since we know that these cells play an important role in the genesis of hypertension. Based on our data provided, α1AMPK might have important functions in this setting. Future research then must determine whether AMPK is involved in the genetic alterations that may influence aging-related cardiovascular diseases, like hypertension and coronary artery disease. Overall, AMPK represents a prime target for future investigation that will result in applicable therapies against inflammation-associated cardiovascular diseases.
